# Associations between socio-spatially different urban areas and knowledge, attitudes, practices and antibiotic use: A cross-sectional study in the Ruhr Metropolis, Germany

**DOI:** 10.1371/journal.pone.0265204

**Published:** 2022-03-10

**Authors:** Dennis Schmiege, Timo Falkenberg, Susanne Moebus, Thomas Kistemann, Mariele Evers

**Affiliations:** 1 Department of Geography, University of Bonn, Bonn, Germany; 2 Institute for Hygiene and Public Health, GeoHealth Centre, University Hospital Bonn, Bonn, Germany; 3 Institute for Urban Public Health, Essen University Hospital, University of Duisburg-Essen, Duisburg, Germany; 4 Center for Development Research, University of Bonn, Bonn, Germany; National University of Science and Technology, ZIMBABWE

## Abstract

Inappropriate and excessive antibiotic use fuels the development of antibiotic resistance. Determinants of antibiotic use, including knowledge and attitudes, are manifold and vary on different spatial scales. The objective of this study was to examine the associations between socio-spatially diverse urban areas and knowledge, attitudes, practices and antibiotic use within a metropolitan city. A cross-sectional survey was conducted in the general population in socio-spatially different areas in Dortmund, Germany, in February and March 2020. Three urban areas were chosen to represent diverse socio-spatial contexts (socio-spatially disadvantaged: A, intermediate: B, socio-spatially disadvantaged: C). Participants were selected via simple random sampling. The questionnaire comprised knowledge and attitude statements and questions around antibiotic use and handling practices. Differences between the areas were examined by estimating odds ratios (OR) and corresponding 95% confidence intervals by multiple logistic regression. Overall, 158 participants were included. Participants of Area C showed the lowest proportions of correct knowledge statements, indicated more often attitudes contrary to common recommendations, lower risk awareness and reported more often antibiotic use (C: 40.8%; A: 32.7%; B: 26.5%) and potential mishandling practices (C: 30.4%; A: 9.6%; B: 17.3%). The multiple logistic regression confirmed these differences. Around 42.3% (C), 33.3% (A) and 20.0% (B) of the diseases mentioned for which an antibiotic was used are mainly caused by viral pathogens. A common misconception across all areas was the perception of antibiotic resistance as an individual rather than a universal issue. This study reveals distinct differences between socio-spatially diverse urban areas within a metropolitan city, regarding knowledge, attitudes and practices around antibiotics and ABR. Our findings confirm that enhanced efforts are required to better inform the population about the adequate use and handling of antibiotics. This study emphasizes the need for future interventions to be tailored to the specific local socio-economic context.

## Introduction

More than 700,000 deaths per year are attributable to drug-resistant infections globally [1], with a projected increase that reaches into the millions in coming decades. Antibiotic resistance (ABR), a natural process whereby bacteria become resistant against antibiotics commonly used to treat infections caused by them [2], is already a serious global health concern. Antibiotic-resistant infections are not just linked to higher mortality, but also associated with higher morbidity, longer hospital stays and higher medical costs [3, 4].

Inadequate and excessive use of antibiotics in humans, animals and plants, have been identified among the key drivers of this “silent pandemic” [5, 6]. Antibiotic consumption in human medicine has increased globally between 2000 and 2015 [7] and varies on different spatial scales. For instance, from between countries differences [8] down to intra-city variations [9, 10], and between health care sectors with the great majority of antibiotics used in the community (i.e. outpatient settings) [11].

Determinants of antibiotic use in the community are manifold, including individual-related (i.e. compositional) and space-related (i.e. contextual and collective) factors [12]. Identifying modifiable determinants on both the supply (e.g. prescriber) and demand (e.g. patients) sides is crucial to improve the appropriate and further reduce antibiotic use. The general population occupies thereby a pivotal role. Among other determinants of antibiotic use, such as the socio-economic status of patients [13–16], knowledge and attitudes towards antibiotic use have also been identified as influencing factors [17].

Educational interventions as one component of multifaceted strategies to tackle ABR were anchored in the World Health Organization’s (WHO) Global Action Plan on Antimicrobial Resistance [18] and subsequently transferred into national action plans, including the German strategy [19]. A systematic review on the effectiveness of interventions to improve awareness and behaviour revealed a notable potential in schoolchildren and parents and less clear evidence for the general public [20]. However, identifying and analysing public knowledge and attitudes on antibiotics and ABR, as well as handling practices are important first steps towards assessing patients’ demands and needs. The resulting insights can be used to inform awareness-raising campaigns and to design effective public health policies to tackle ABR.

Previous knowledge, attitude and practice (KAP) studies have focused on various population groups in different countries, e.g. the general population [21–28], (medical) students [29–32], parents [33], pilgrims [34] or pharmacists and physicians [35, 36]. As antibiotic consumption and its determinants show spatial variation, patients’ demands and needs also vary between and within countries, assumingly also on intra-city levels, e.g. between different neighbourhoods. However, research, particularly on this geographical aspect and differences between socio-spatially diverse urban areas, is scarce.

In Germany, dispensing volumes of antibiotics were higher in veterinary as compared to human medicine in 2016 [37] but have significantly decreased since, now being on comparable levels [38]. Regarding human medical treatment, around 85% of antibiotics were used in the outpatient sector [39] with spatial differences down to the city level (data from [40]). However, there is a paucity of KAP research on antibiotics and ABR in Germany and on intra-city differences. One study in the federal state Lower Saxony identified good knowledge on antibiotics but limited knowledge on ABR, multi-drug resistant pathogens, and their consequences [23]. Limited knowledge on the application of antibiotics, inappropriate patient expectations, as well as a discrepancy between knowledge and action, were also highlighted by another survey among 3,100 German-speaking persons [41].

The rationale of this study was therefore twofold. First, contributing to closing the knowledge gap on KAP regarding antibiotics and ABR in Germany and secondly, examining the associations between KAP, antibiotic use and socio-spatially diverse urban areas. The household survey aimed to assess knowledge and attitudes on antibiotics and ABR, as well as self-reported antibiotic use and handling practices in the general population of three socio-spatially different sub-districts within one city in the Ruhr Metropolis, Germany. This approach allows for the identification of common misconceptions (i.e. across all areas) and potential differences between diverse urban areas and thereby enable more tailored educational or behavioural interventions.

## Material and methods

This study was designed as a cross-sectional, observational study using a structured questionnaire in the adult general population in the city of Dortmund, Germany. The reporting of this study follows the STROBE (Strengthening the Reporting of Observational Studies in Epidemiology) statement [42]. Tablet-based face-to-face interviews were conducted in the German language in February and March 2020 mainly on weekdays. One weekend day was chosen in addition in each area to reduce sampling bias. Study participants were interviewed in their homes. For this publication, statements were translated into English. All study participants were older than 18 years. Before the interview, they were informed about the nature of the study and provided written informed consent. The Ethics Commission of the medical faculty of the University of Bonn approved this study (registration number: 052/20).

### Selection of study areas and sampling procedure

The city of Dortmund is the most populated city in the Ruhr Metropolis with distinct social and ethnic segregation that also translates into health-related environmental inequalities [43]. It ranked second in antibiotic use among the 26 cities and municipalities in the region in 2019 (ATC group J01; data from [40]).

**Fig 1:**
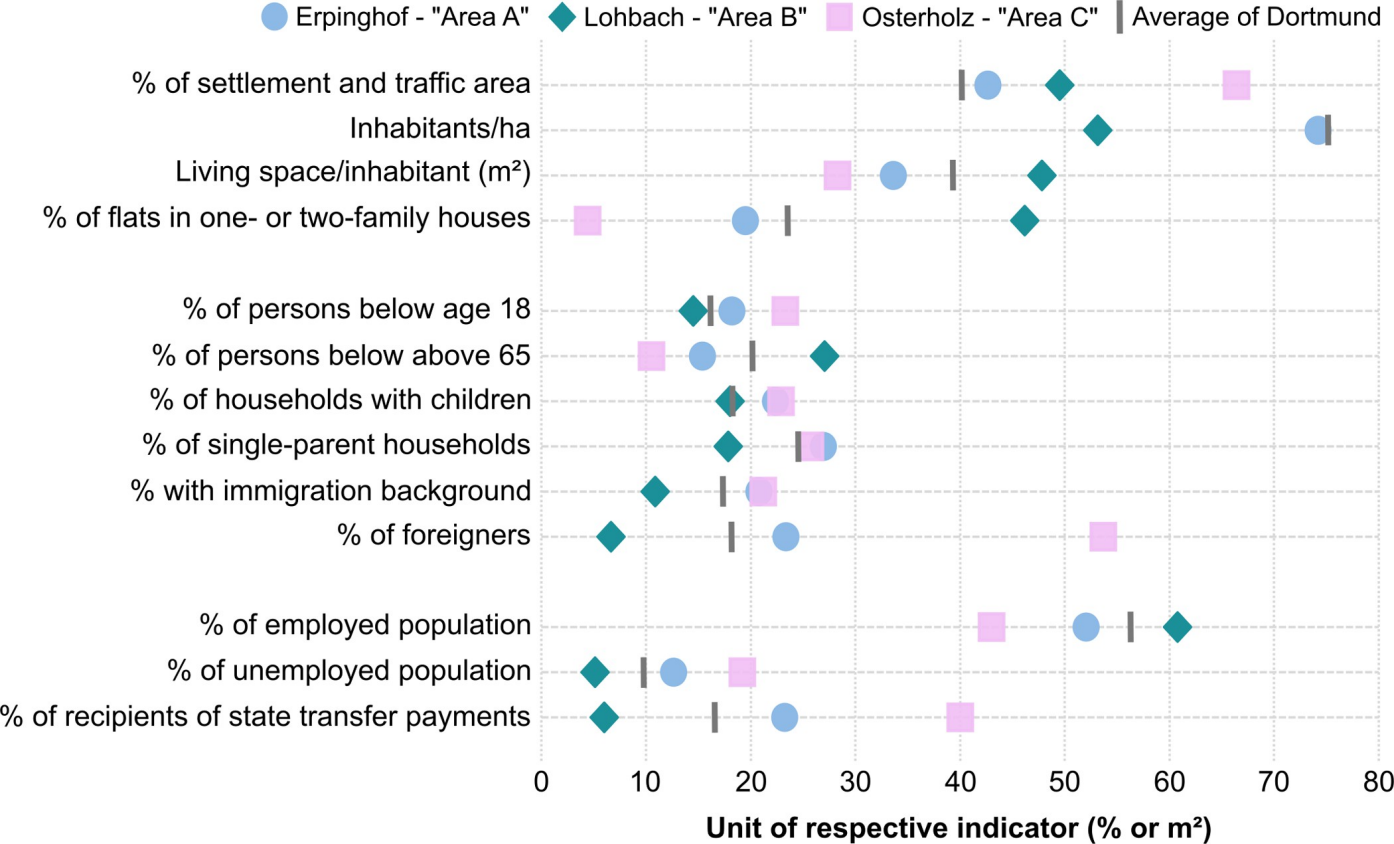
Socio-spatial structure of the three selected study areas (data source: Stadt Dortmund, 2019).

In a previous study in the German population, age, immigration status and self-assessed social status were associated with limited health literacy [44]. Accounting for this and also the socio-economic north-south gradient of the city [45], a multistage sampling approach was used. In the first stage, three urban areas (i.e. Erpinghof, Lohbach and Osterholz) were chosen based on previous studies [46–48] to represent distinct socio-spatial contexts. Fig 1 displays the differences of socio-spatial indicators between the areas (for detailed information on the indicators see S1 Table). For easier reference, the areas are referred to as Erpinghof–“Area A”, Lohbach–“Area B” and Osterholz–“Area C”.

Differences are particularly observable between Area B and Area C whereas Area A is often located in between those. Area B exhibits a lower share of settlement and traffic area, the lowest population density, the highest living space per inhabitant, and the highest share of flats in one- or two-family houses. It also displays the lowest shares of households with children or foreigners. In terms of socio-economic indicators, the shares of the unemployed population and recipients of state transfer payments are lowest in Area B. Overall, those indicators point towards a comparatively socio-spatially advantaged situation in Area B compared to the other two areas.

In a second step, residential buildings in the three areas were randomly selected. Shapefiles containing all buildings in the respective areas were downloaded (TIM-online, https://www.tim-online.nrw.de/tim-online2/) and prepared for the sampling procedure by selecting only buildings with the official function “residential house”. These steps were implemented in QGIS [49]. Accounting for the different shares of flats in one- and two-family houses between the three areas (see Fig 1), the number of buildings sampled was higher in Area B (300 compared to 200 in Areas A and C). All households in a selected residential building were considered eligible for participation. The study population encompassed all adults (above 18 years) living in one of the three socio-spatially different areas in the city of Dortmund.

Announcement flyers (not revealing the actual topic of the survey to avoid introducing bias) were distributed two weeks before the survey to allow selected households to withdraw before being approached.

### Questionnaire

The theoretical framework of this study and the development of the questionnaire were both informed by the KAP model, which postulates that increasing a person’s knowledge will prompt a behaviour change [50]. The structured questionnaire consisted of four parts: i) knowledge of antibiotics and ABR, ii) attitudes towards antibiotic use and risk awareness of ABR, iii) handling practices and antibiotic use, and iv) demographic standards. Previously tested and used questions were selected from other KAP studies [21, 22, 29, 51–53] to ensure comparability. The questionnaire was discussed and refined in different research groups and pre-tested with a few people outside of academia to ensure comprehensibility and determine its duration.

Knowledge on antibiotics and ABR were inquired with nine statements that were read to the participants and to which they were asked to indicate whether they are correct, false or “Don’t know”. Regarding antibiotics, knowledge was assessed based on five statements covering aspects of efficacy against bacteria and viruses, possible medical indication for the flu and common colds and urinary tract infections (UTI), as well as side effects of antibiotics. For ABR, knowledge statements covered aspects around the consequences of over- and misuse of antibiotics, the interconnectedness of agriculture and human medicine, and the potential consequences of ABR.

Attitudes towards antibiotics and risk awareness of ABR were investigated via five statements each to which participants were asked to respond on a five-point Likert scale from “strongly disagree” to “strongly agree”. Attitudes towards antibiotics included the following aspects: behaviour when sick with flu or a common cold and requesting information from the physician when no antibiotic is prescribed, termination of antibiotic treatment when feeling better, keeping antibiotics at home, and passing on antibiotics to relatives or friends. Regarding the risk awareness of ABR, study participants were asked about ABR as an issue on different spatial scales from the global to the family and individual level, ABR as an issue only for those that take antibiotics, and future effectiveness of antibiotics against the same disease.

Inquiring handling practices of antibiotics, study participants were asked whether any household member has ever used an antibiotic to filter out those that never used any. Three questions on their handling practices followed covering the source of antibiotics, general treatment adherence, and disposal of antibiotics. For each question, interviewees could choose multiple times from a pre-determined list of answers. For all statements, questions and corresponding answers in German and English language see S1 File (Part A).

Allowing for socio-economic detailed analyses, the demographic items were assessed, including age, gender, origin, civil status, education, training, employment situation, occupational sector, participant’s and household net income, religious beliefs and health insurance. The questions are based on the German Federal Statistical Office [54].

### Data analysis

All statistical analyses were implemented using R (version 4.1.0, [55]). Multiple logistic regression was used to estimate odds ratios (OR) and corresponding 95% confidence intervals (CI). Outcomes of interest were low knowledge, attitudes contrary to common recommendations, lower risk awareness, potential mishandling or antibiotic use. The outcome variables and covariates including their respective coding are shown in S1 File (Part B). The minimal sufficient adjustment sets were derived using directed acyclic graphs [56, 57]. Univariate ORs for the area variable were adjusted for confounding by including the following variables in the multivariate analysis: age, immigration background, family status and household income.

In case of a high prevalence of the outcome in cross-sectional studies, the estimation of prevalence ratios (PR) should be preferred because ORs can have some limitations (e.g. overestimation) [58–60]. However, problems of convergence are a known issue of log-binomial models used to estimate PRs [61], which was also encountered in this study when adjusting for confounders. Following Zocchetti et al. [62], the prevalence of outcomes and exposures in this study are in the majority of cases within a value range in which the overestimation by OR is tolerable. As the focus of our analyses was more on the direction of the effect estimators, it was deemed justified to use ORs to estimate associations.

## Results

The sampled buildings in the three study areas contained 2,396 possibly accessible household units (i.e. no vacancy, functional doorbells) of which 1,382 could be contacted. In total, 158 interviews were conducted before the household survey had to be cancelled prematurely in mid-March 2020 due to the COVID-19 pandemic. This marks a response rate of 11% (158/1,382; Area A: 12% (52/434); Area B: 16% (50/305); Area C: 9% (56/643)). Study participants were almost equally distributed between the three areas. Table 1 illustrates the demographic and socioeconomic characteristics of the interviewees.

**Table 1:** Demographic and socioeconomic indicators of the study participants grouped by area.

Indicator		Area A	Area B	Area C
		(n = 52)	(n = 50)	(n = 56)
		n (%)	n (%)	n (%)
Age	Median [Q1-Q3]	48.5	63	30
		[35.8–63.0]	[50.0–70.0]	[23.5–41.5]
Gender	Female	29 (55.8)	24 (48.0)	25 (44.6)
	Male	23 (44.2)	26 (52.0)	30 (53.6)
	Diverse	0 (0.0)	0 (0.0)	1 (1.8)
Family status	No partnership	17 (32.7)	18 (36.0)	36 (64.3)
	In a partnership	35 (67.3)	32 (64.0)	20 (35.7)
Origin	German	35 (67.3)	40 (80.0)	19 (33.9)
	Immigrant or descendant of immigrant	17 (32.7)	10 (20.0)	37 (66.1)
Education	Secondary	9 (17.3)	5 (10.2)	20 (37.0)
	Post-secondary non-tertiary	29 (55.8)	19 (38.8)	15 (27.8)
	Tertiary	14 (26.9)	25 (51.0)	19 (35.2)
Income	Median group (€)	1500–1999	2000–2499	1000–1499
	Below the national average	29 (56.9)	17 (36.2)	46 (85.2)
	Equal to or above the national average	22 (43.1)	30 (63.8)	8 (14.8)
Occupational sector	Health and social	9 (17.3)	14 (28.0)	15 (26.8)
	Other	43 (82.7)	36 (72.0)	41 (73.2)

Percentages may not add up to 100% because of rounding. Missing values occurred for age, education and income but were overall very low (max. n = 3 for income in Area B).

Characteristics of the study participants varied profoundly between the three areas with Area B and Area C often showing opposing situations. Compared to Area B, study participants in Area C were younger, less often in a partnership, more often immigrants or descendants of immigrants, had lower education and reported more often incomes below the national average. Except for the indicator gender, characteristics of study participants in Area A were usually positioned between Areas B and C. The distribution of demographic and socio-economic indicators of the study participants between the three areas mirrors the socio-spatial situation indicated by the official city statistics (see Fig 1).

### Spatial variation of knowledge on antibiotics and antibiotic resistance

**Fig 2:**
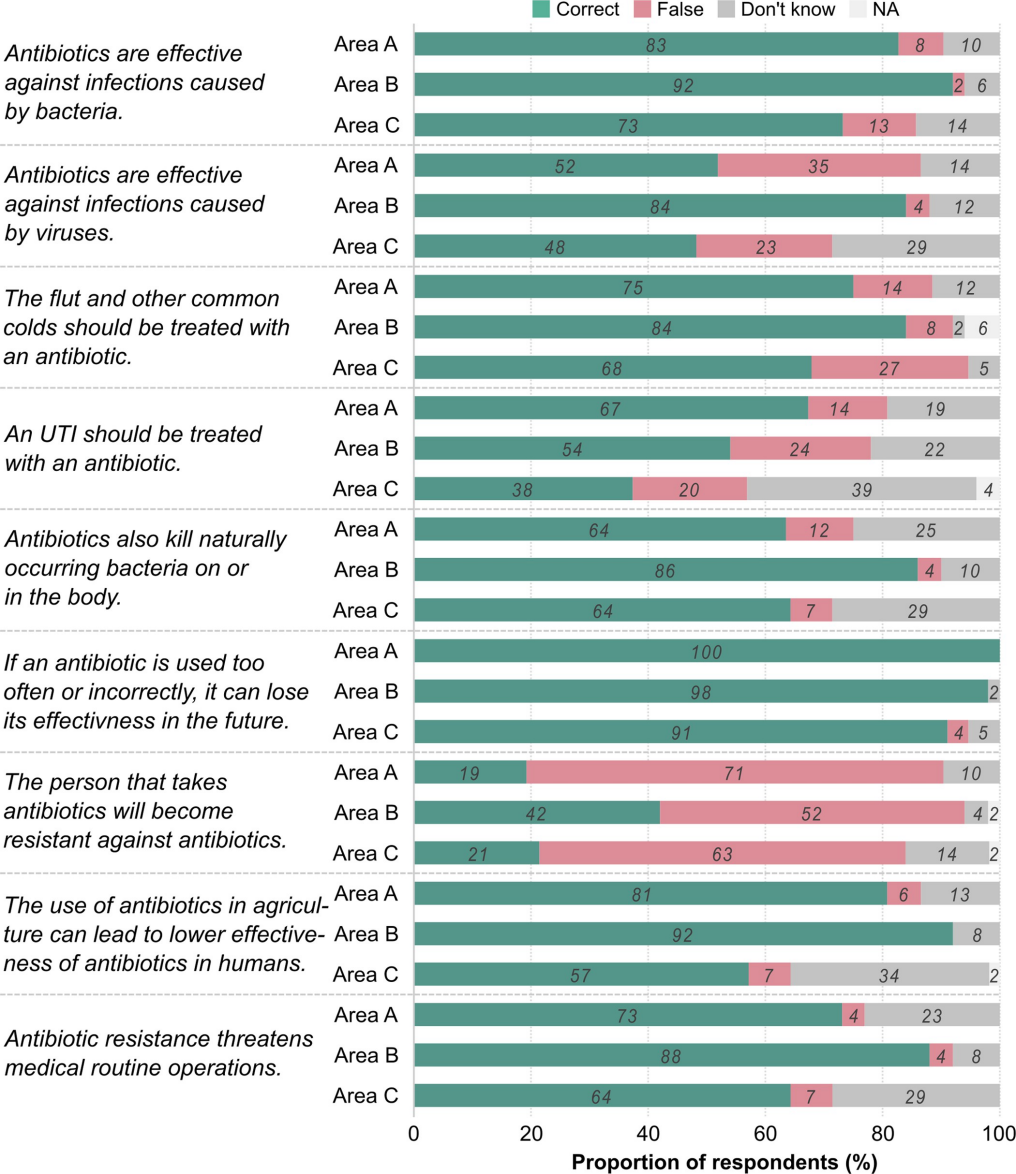
The proportion of study participants replying to the knowledge statements grouped into the three areas. Area A: n = 52; Area B: n = 50; Area C: n = 56. UTI–urinary tract infection. Statements were re-coded that rightly stating a statement was false is shown as “correct”.

Participants were asked to indicate for nine knowledge statements whether they are correct or false. Fig 2 illustrates the proportion of interviewees answering the statements correctly segregated by research area. If respondents stated rightly that a statement was false, this was re-coded as answering the statement correctly for this figure.

For the majority of statements, more than half of the respondents answered correctly. Study participants in Area B showed the highest proportions of correct answers to most knowledge statements (except for the UTI and the future effectiveness statements). On the contrary, interviewees in Area C often displayed the lowest proportions. The proportion of respondents answering correctly in Area A was often between the other two areas.

Knowledge of the majority of study participants on the effectiveness of antibiotics against bacteria was better as compared to viruses. Regarding indications for antibiotic use, most interviewees in all areas knew that antibiotics are not indicated for flu or common colds. However, certainty among respondents was much lower for UTIs indicated by higher proportions of “Don’t know”. More than two-thirds of study participants were aware of side effects, the connection to the agricultural sector and possible consequences of ABR. The great majority of study participants in all areas answered correctly about the future effectiveness of antibiotics. A common misconception across all areas was that people (and not bacteria) would become resistant to antibiotics. Table 2 presents the association between the area variable and respective eight knowledge statements.

**Table 2:** Association between false knowledge statements and urban areas (reference: Area C).

	Area A		Area B	
Knowledge statement	Crude OR	Adjusted OR^a^	Crude OR	Adjusted OR^a^
	[95% CI]	[95% CI]	[95% CI	[95% CI]
*Effective against bacteria*	0.57	0.54	**0.24**	0.27
	[0.22–1.43]	[0.16–1.70]	[0.06–0.72]	[0.05–1.15]
*Effective against virus*	0.86	1.03	**0.18**	**0.24**
	[0.40–1.84]	[0.40–2.64]	[0.07–0.43]	[0.07–0.74]
*Antibiotic use indicated for flu*	0.70	1.09	**0.25**	0.53
	[0.30–1.62]	[0.40–2.96]	[0.08–0.70]	[0.13–2.00]
*Antibiotic use indicated for UTI*	**0.31**	**0.34**	0.54	0.64
	[0.14–0.68]	[0.13–0.85]	[0.25–1.18]	[0.22–1.83]
*Side effects of antibiotic use*	1.04	0.83	**0.29**	**0.16**
	[0.47–2.28]	[0.31–2.15]	[0.10–0.74]	[0.04–0.56]
*Person becomes resistant*	1.17	1.73	**0.37**	0.48
	[0.46–3.06]	[0.59–5.26]	[0.15–0.86]	[0.15–1.48]
*Connection to agricultural sector*	**0.33**	0.66	**0.12**	0.42
	[0.13–0.78]	[0.23–1.90]	[0.03–0.35]	[0.09–1.64]
*Threat to medical operations*	0.66	1.22	**0.25**	0.49
	[0.29–1.50]	[0.46–3.30]	[0.08–0.64]	[0.13–1.68]

^a^ Adjusted for age, immigration background, family status and household income; UTI–urinary tract infection; OR >1 indicates an increased chance of replying incorrectly; the OR for the future effectiveness statement could not be calculated due to very low numbers of false replies.

Adjusted ORs for replying falsely to the knowledge statements were consistently lower in Area B indicating higher knowledge. The differences between Areas C and A were less clear pronounced. Whereas for some knowledge statements adjusted ORs were lower, they were higher for others. Three statements ranged closely around one revealing a rather comparable situation between those two areas.

### Spatial variation of attitudes towards antibiotics and antibiotic resistance

**Fig 3:**
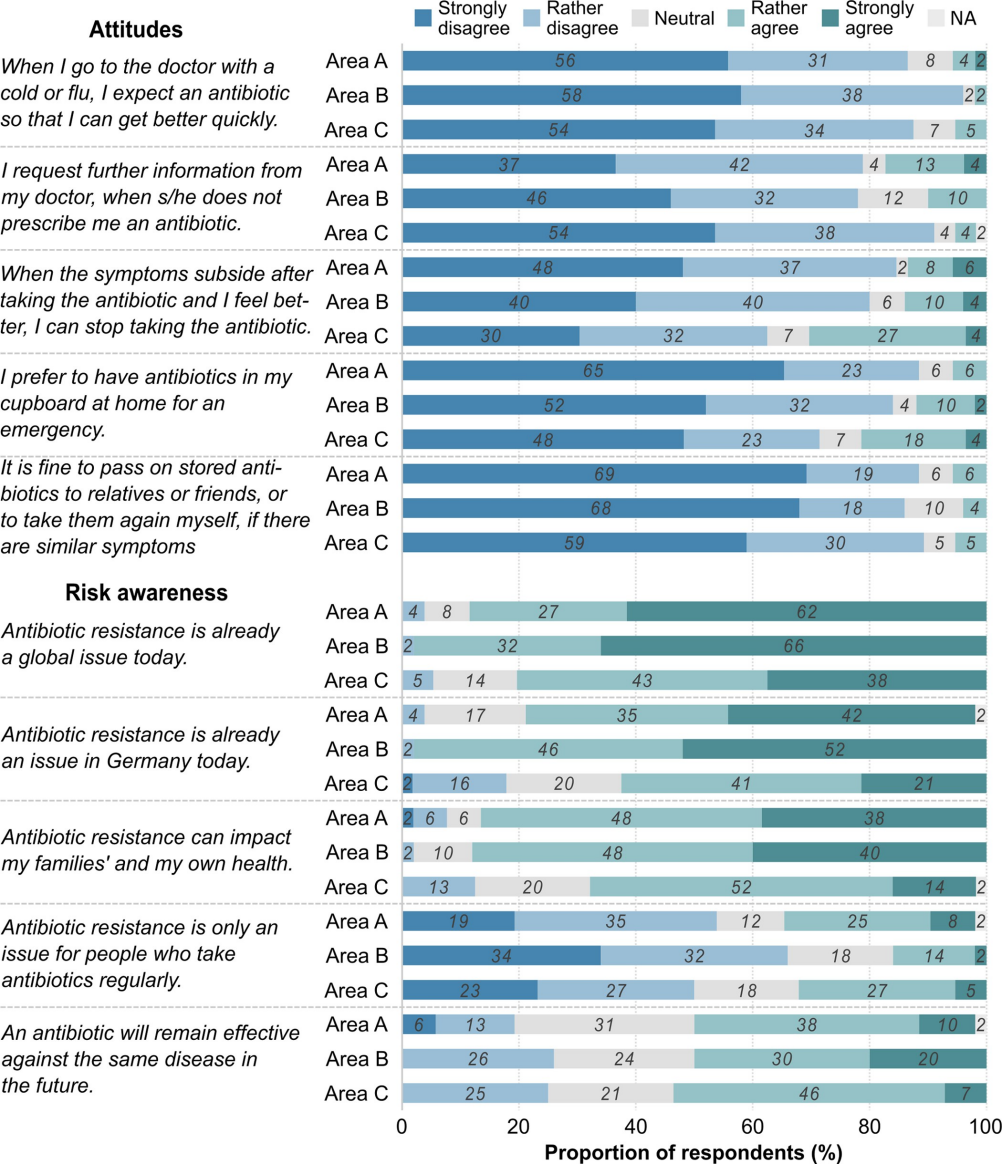
The proportion of study participants replying to the attitude statements grouped into the three areas. Area A: n = 52; Area B: n = 50; Area C: n = 56.

Attitudes towards antibiotics and risk awareness of ABR were queried via five statements each. Fig 3 depicts the attitudes of study participants towards antibiotics and ABR in the three areas.

The great majority of study participants rather or strongly disagreed with the statements on expectations to receive an antibiotic when visiting the doctor because of flu or a cold, requesting further information from the doctor in the absence of an antibiotic prescription, as well as sharing behaviour or re-use of antibiotics. Only for the statements on treatment adherence and storage willingness, some participants revealed attitudes contrary to common recommendations. About 14% of respondents in Area A and Area B, as well as 27% in Area C rather or strongly agreed that they could stop taking an antibiotic once they feel better. Around 22% of interviewees in Area C rather or strongly agreed that they prefer to store antibiotics at home.

The majority of study participants considered ABR already a global issue today. The numbers slightly declined when moving from the global level to Germany and remained stable when considering ABR as an issue at the family level. About 16% (Germany) and 13% (family) of interviewees in Area C rather or strongly disagreed with these statements. More than half of respondents in all areas rather or strongly agreed that ABR is only an issue for persons who take antibiotics regularly. Comparable to the knowledge statement, this presents an apparent misconception across areas. Half of the study participants in each area rather or strongly agreed that antibiotics will retain their effectiveness in the future. Table 3 illustrates the association of the area variable with the ten attitude statements.

**Table 3:** Association between attitudes contrary to common recommendations or low risk awareness and urban areas (reference: Area C).

	Area A		Area B	
	Crude OR	Adjusted OR^a^	Crude OR	Adjusted OR^a^
	[95% CI]	[95% CI]	[95% CI	[95% CI]
**Attitudes**				
*Expect an antibiotic*	1.09	1.99	0.29	0.87
	[0.35–3.42]	[0.54–7.69]	[0.04–1.28]	[0.10–5.30]
*Request further information*	**3.42**	1.61	**3.60**	1.45
	[1.08–13.07]	[0.42–6.95]	[1.13–13.77]	[0.32–7.10]
*Discontinue when symptoms subside*	**0.30**	0.50	**0.42**	0.79
	[0.11–0.74]	[0.17–1.39]	[0.17–0.98]	[0.23–2.66]
*Have antibiotics at home*	**0.33**	0.65	0.48	2.17
	[0.11–0.88]	[0.19–2.12]	[0.18–1.21]	[0.53–9.60]
*Pass on antibiotics*	1.09	2.51	1.36	4.27
	[0.32–3.71]	[0.61–11.50]	[0.42–4.51]	[0.88–23.32]
**Risk awareness**				
*ABR as global issue*	0.53	0.83	**0.08**	0.28
	[0.17–1.53]	[0.22–3.04]	[0.00–0.45]	[0.01–2.34]
*ABR as issue for Germany*	0.46	1.14	**0.03**	**0.15**
	[0.19–1.07]	[0.38–3.45]	[0.00–0.17]	[0.01–0.98]
*ABR can impact family and own health*	**0.32**	0.43	**0.28**	0.54
	[0.11–0.82]	[0.13–1.29]	[0.09–0.74]	[0.13–1.99]
*Only issue for people taking antibiotics*	0.82	1.60	0.52	1.06
	[0.38–1.76]	[0.61–4.32]	[0.23–1.12]	[0.34–3.35]
*Antibiotics will remain effective in future*	1.20	1.68	1.15	1.41
	[0.56–2.58]	[0.66–4.39]	[0.54–2.49]	[0.49–4.15]

^a^ Adjusted for age, gender, immigration background, family status, household income and occupational sector; ABR: antibiotic resistance; OR >1 indicates increased chance of replying contrary to common recommendations (attitudes) and lower risk awareness (risk awareness).

Study participants in Area A displayed greater adjusted OR for expecting an antibiotic. Living in Areas A and B was associated with greater adjusted OR for requesting further information and passing on antibiotics to relatives or friends. On the contrary, interviewees in both areas showed lower adjusted OR for discontinuing the antibiotic treatment when the symptoms subside.

Overall, living in Area B was associated with consistently lower adjusted ORs of perceiving ABR as a global issue, for Germany and at the family level. Study participants in Area A, on the other hand, showed similar or greater adjusted ORs (except for ABR impact on families’ and own health). ORs were similar or greater in Areas A and B for considering ABR an issue only for those people that take antibiotics and the future effectiveness of antibiotics.

### Spatial variation of handling practices of antibiotics and self-reported antibiotic use

Antibiotic handling practices, including the source of antibiotics, general treatment adherence, and disposal, were assessed via three questions (see S1 Fig). Around 87% of participating households have ever used an antibiotic, most of them prescribed from a physician (inpatient and/or outpatient). Only one respondent in Area B and three in Area C indicated that they used an old package. Most of the respondents either followed the doctor’s instructions or used the package completely but some reported using an antibiotic until they feel better (Area A: 2.9%, Area B: 3.2%, Area C: 14.8%). Regarding the disposal, participants mentioned most often to consume all antibiotics, return the package to the pharmacy and/or dispose of in the domestic or special waste. Respondents in each area also indicated storing antibiotics at home (Area A: 6.7%, Area B: 11.1%, Area C: 21.4%). None of the interviewees disposed of antibiotics into the toilet, which is much lower as identified in another survey in Germany (15%) [63]. The statistical analysis of the three reported possible mishandling practices (i.e. using an old package, stop treatment when feeling better and storing antibiotics at home) revealed lower adjusted OR in Area A (0.40, 95% CI: 0.11–1.29) and greater OR in Area B (1.58, 95% CI: 0.44–5.96) compared to Area C.

One-third of the participants (49) reported antibiotic use within the last 12 months (i.e. March 2019-March 2020) amounting to 69 antibiotic treatments (including household members: 95 people and 151 treatments). Self-reported antibiotic use of the interviewees followed a seasonal trend with increasing reported consumption in autumn and the highest values in winter months (47.9% of all mentions). Spatially, most antibiotic use was reported in Area C (40.8%), followed by Area A (32.7%) and Area B (26.5%), translating in adjusted ORs of 0.44 (95% CI: 0.16–1.21) and 0.80 (95% CI: 0.26–2.50) compared to reference Area C. Antibiotics were prescribed in 42.3% (Area C), 33.3% (Area A) and 20.0% (Area B) of the cases for diseases, which are predominantly caused by viral pathogens (i.e. cold, flu and pharyngitis).

## Discussion

This study reveals overall a relatively good knowledge, attitudes that can be evaluated positively, high risk awareness and low mishandling with distinct spatial variation between the three socio-spatially different areas.

The proportions of interviewees answering correctly to the knowledge statements are within similar value ranges compared to other studies in the general population in European countries [21, 23, 25, 51, 64] but consistently higher as opposed to studies from non-European middle- and high-income countries [22, 24, 26, 29, 34, 65].

The great majority of study participants replied according to common recommendations for each attitude statement. Attitudes contrary to common recommendations in this study included stopping the antibiotic treatment when the participant felt better and the preference to having antibiotics stored at home, both particularly prevalent in Area C. Proportions were slightly lower as in a study from Sweden [21] but often much higher as found in studies in Kuwait [22], Lebanon [24] and Saudi Arabia [34].

This study reveals that there is a need to inform people on the adequate use of antibiotics. Almost 40% of respondents did not reply correctly about the efficacy of antibiotics against viruses, which is slightly higher compared to the other German KAP study [23]. It is striking that around one-third of the disease mentions against which an antibiotic was reportedly taken are mainly caused by viral pathogens. Acknowledging that there are circumstances in which an antibiotic becomes necessary for one of those diseases (or attendant symptoms), this finding still points towards potentially misused antibiotics in the study population, which was also identified in a previous survey in Germany [41]. It is further necessary to inform people about the correct handling of antibiotics with an emphasis on treatment adherence and disposal (i.e. not storing antibiotics at home), particularly in Area C.

### The misconception of antibiotic resistance as an individual issue

The majority of study participants considered ABR as a global issue already today, as well as at the national (Germany) and individual (family and own health) levels. Albeit this tendency, many also indicated that ABR is an individual issue and only for people who take antibiotics regularly. This opinion was prevalent across all three areas revealing a common misconception, which was also identified in other surveys [21, 22, 53, 66]. It highlights the apparent lack of understanding that ABR is a universal issue that can affect everyone, even if the person did not take an antibiotic. Tackling this, re-framing the messaging (e.g. in information campaigns) by focusing on a sense of personal jeopardy and using human stories and thereby emphasizing the personal relevance was proposed as a way forward [67].

### Differences between socio-spatially diverse urban areas

Albeit knowledge, attitudes, risk awareness and handling practices were overall fairly well, differences between the three socio-spatially diverse urban areas could be identified pointing towards an unfavourable situation in the socio-spatial disadvantaged area (Area C). Similar differences between affluent and deprived areas were also observed in Greater London [35].

The knowledge statements revealed a clear spatial trend with the lowest knowledge in Area C and the highest proportions of participants answering correctly consistently in Area B (one exception: medical indication for UTI). Attitudes and risk awareness between the three areas were more differentiated but still highlighted some spatial tendencies with higher risk awareness in Area B. Potential mishandling practices were most prevalent in Area C but the OR of engaging in such behaviour was higher in Area B. Summarizing, study participants in Area C were less knowledgeable, displayed lower risk awareness and reported more often mishandling practices and antibiotic use whereas participants in Area B usually presented the opposite situation. Interestingly, the occurrence of multidrug-resistant bacteria in urban wastewater sampled from the identical areas revealed the same patterns with higher values in Area C and lower resistance levels in Area B [68].

The population structure partly explains the variation of knowledge, attitudes, risk awareness and handling practices between the three urban areas. However, adjusting for those compositional factors, differences in ORs between the three areas remained, highlighting the existence of other unaccounted for determinants, e.g. possible influences of contextual (i.e. opportunity structures in the local physical and social environment) and collective (i.e. socio-cultural features) factors [69], which require further investigation.

This is the first study to focus explicitly on differences between socio-spatially diverse urban areas relating to KAP on antibiotics and ABR in Germany. Even after controlling for relevant confounders, differences between the areas prevailed underlining the robustness of the results. Albeit the premature cancellation of the survey and the relatively low response rate both resulting in a relatively small sample size, the study population in the three areas still mirrors the situation determined by official statistics. However, the generalisation of findings from this survey to other national and international cities still needs to be validated. Further limitations deserve mentioning. We did not use a validated questionnaire to assess knowledge and attitudes. For instance, it would have been beneficial to ask respondents if they knew what an antibiotic is. Recall bias regarding self-reported antibiotic use may affect the results, which is why we used the meteorological seasons instead of months for reporting. Using OR instead or PR may overestimate the effect when the outcome is highly prevalent. However, only the direction of the adjusted effect estimator was of interest in the statistical analyses.

## Conclusions

This study demonstrates differences between three socio-spatially different areas in a large city in western Germany, regarding knowledge, attitudes, practices and antibiotic use. Knowledge and attitudes on antibiotics and ABR showed distinct spatial differences. Participants of the socio-spatially disadvantaged area (C) were less knowledgeable, had lower risk awareness and reported more often antibiotic use and mishandling practices. The results of this survey, however, need to be validated by quantitative and particularly qualitative research in different population groups and regions. These results can function as a starting point for potential educational interventions. Our results indicate that population-based interventions should be tailored to the specific characteristics (e.g. knowledge, needs, etc.) typical to different socio-economic urban areas to unfold their full potential in informing the public about their individual space for action regarding the global health threat of ABR.

## Supporting information

S1 Fig Figures for self-reported antibiotic use and handling practices. (DOCX)

S1 Table Definitions of indicators used for study area selection. (DOCX)

S1 File All statements, questions and corresponding reply options (in German and English language). (DOCX)
