# Supplementary material for: Associations between socio-spatially different urban areas and knowledge, attitudes, practices and antibiotic use: A cross-sectional study in the Ruhr Metropolis, Germany
Source: PLoS One. 2022 Mar 10;17(3):e0265204. doi: 10.1371/journal.pone.0265204 (PMC8912211; doi:10.1371/journal.pone.0265204)
Supplement: S1 Fig — (DOCX) [file pone.0265204.s001.docx]

**S1 Fig**

**Figures for self-reported antibiotic use and handling practices**


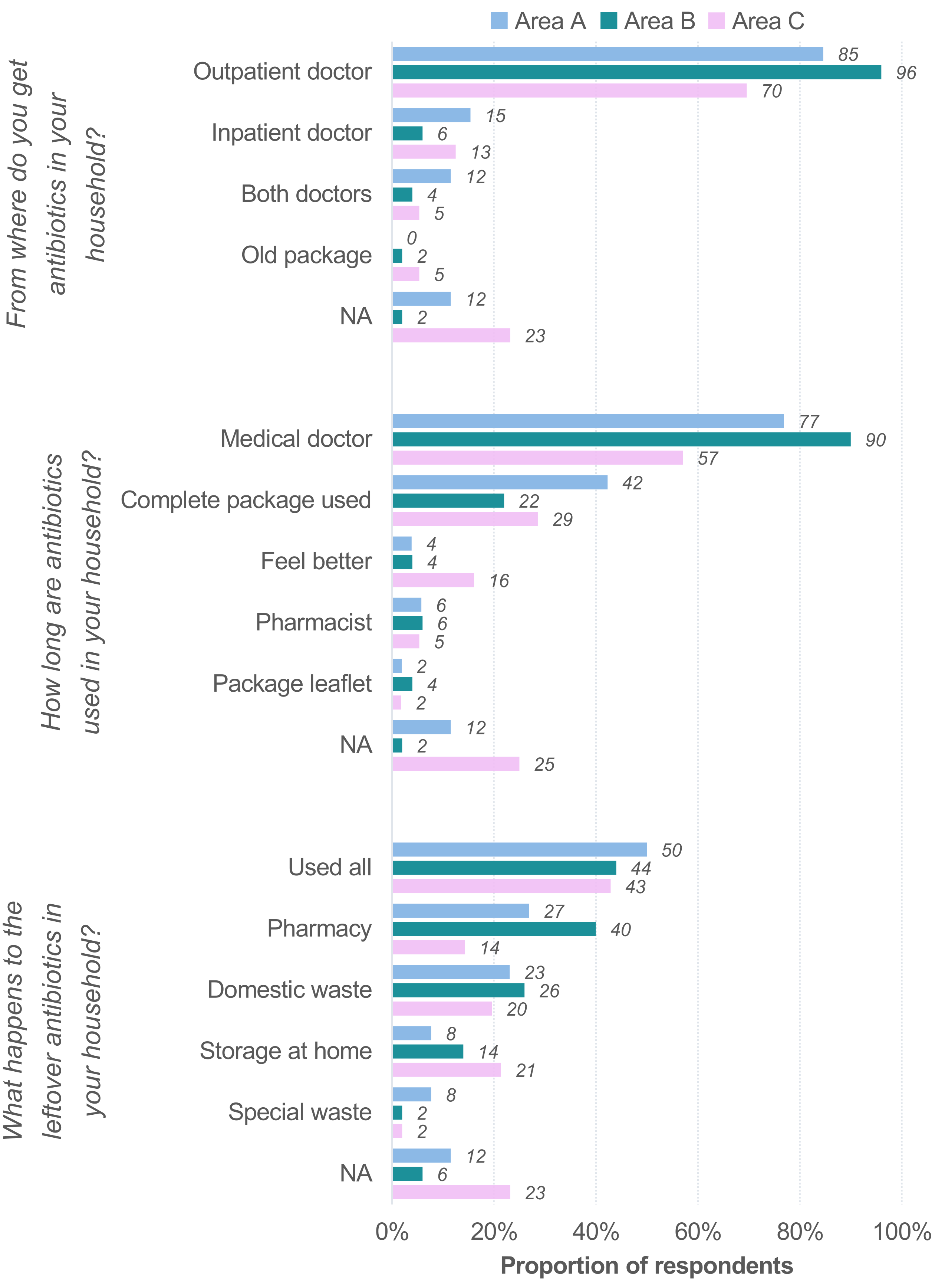


**Figure A.** Handling practices with antibiotics segregated by research areas


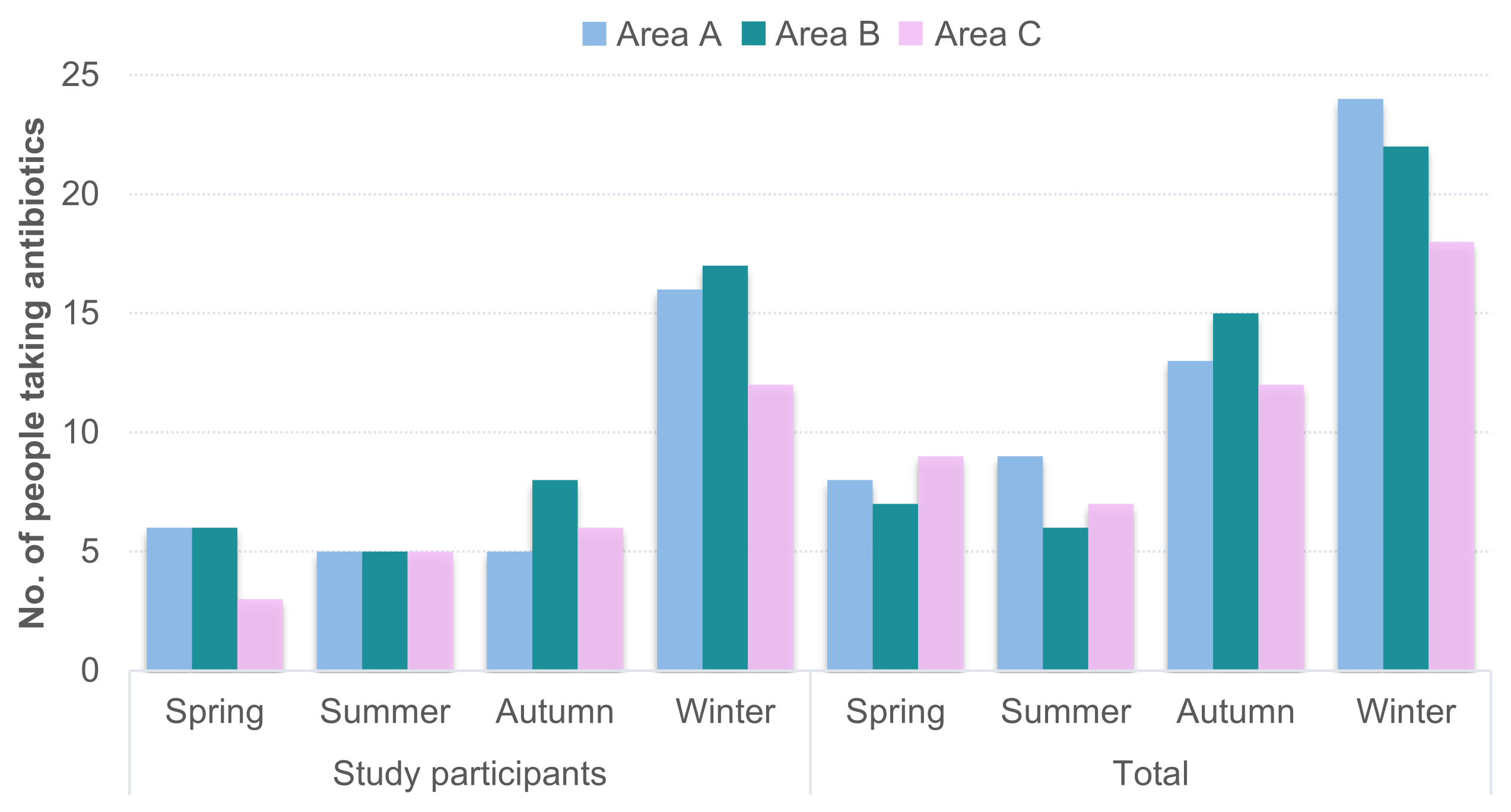


**Figure B.** Self-reported antibiotic use by the study participants (left) and total (right; study participants plus household members) segregated by research area and meteorological season


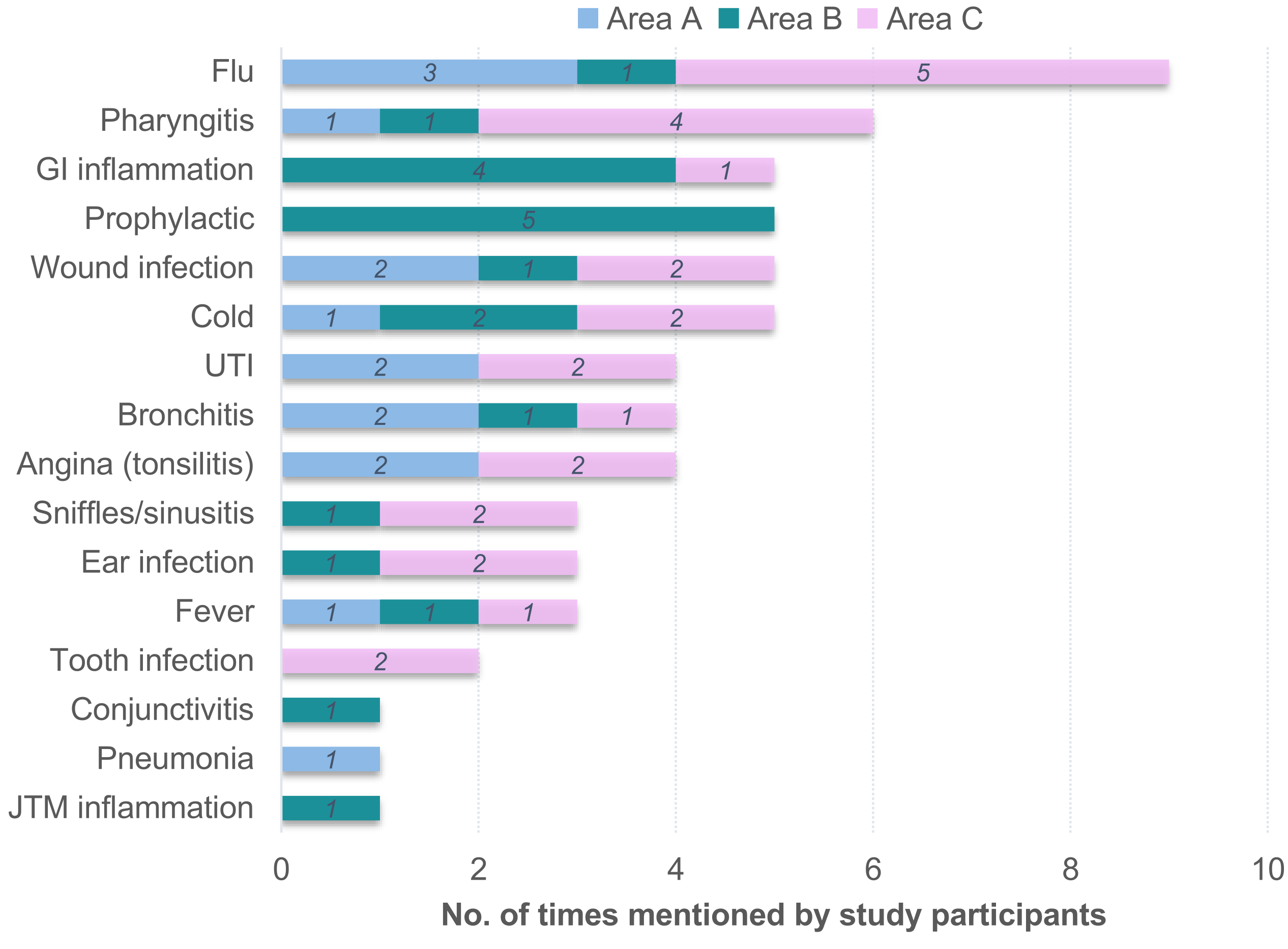


**Figure C.** Diseases mentioned by study participants against which an antibiotic was taken segregated by research area
