# Supplementary material for: Associations between socio-spatially different urban areas and knowledge, attitudes, practices and antibiotic use: A cross-sectional study in the Ruhr Metropolis, Germany
Source: PLoS One. 2022 Mar 10;17(3):e0265204. doi: 10.1371/journal.pone.0265204 (PMC8912211; doi:10.1371/journal.pone.0265204)
Supplement: S1 Table — (DOCX) [file pone.0265204.s002.docx]

**S1 Table.** Definitions of indicators used for study area selection

| **Indicator** | **Full name in reference** | **Pages** | **Additional information** |
| --- | --- | --- | --- |
| Settlement and traffic area | Share of settlement and traffic area of the total area 2018 (%) | 10-11 | -- |
| Inhabitants/ha | Inhabitants (main residential population) as of 31.12.2018 per hectare of settlement area | 16-17 | -- |
| Living space/inhabitant | Living space per inhabitant (population entitled to reside) in buildings with residential space on 31.12.2016 (sqm) | 94-95 | -- |
| Share of flats in one- or two-family houses | Share of apartments in one- and two-family houses in all apartments in residential buildings as of Dec. 31, 2016 (%) | 90-91 | -- |
| Share of persons below age 18 | Percentage of 0- to under-6-year-olds in the main resident population as of Dec. 31, 2018 (%)  +  Percentage of 6- to under-18-year-olds in the main resident population as of Dec. 31, 2018 (%) | 20-21  +  22-23 | -- |
| Share of persons above age 65 | Percentage of 65- to under-80-year-olds in the main resident population as of Dec. 31, 2018 (%)  +  Percentage of 80-year-olds and older in the main resident population as of Dec. 31, 2018 (%) | 30-31  +  32-33 | -- |
| Share of households with children | Share of households with children under 18 in all households as of 12/31/2018 (%) | 84-85 | -- |
| Share of single-parent households | Percentage of single-parent households among all households with children under 18 on Dec. 31, 2018 | 86-87 | -- |
| Share with migration background | Persons with German citizenship and migration background as a percentage of the main resident population in 2018 (%) | 64-65 | Persons with a migration background include*:   - Foreigners and their children - Naturalized persons and their children - (Late) emigrants and their children   **Basis for the assignment is the MigraPro method in which migration background is approximately derived from the population register.* |
| Share of foreigners | Persons with exclusively non-German citizenship as a percentage of the main resident population in 2018 (%) | 66-67 | -- |
| Share of employed population | Proportion of employees subject to social security contributions (at place of residence) in the population aged 18 to under 65 in December 2018 (%) | 100-101 | -- |
| Share of unemployed population | Unemployed registered with the Federal Employment Agency as a percentage of the labor force (employed + unemployed) in December 2018 (%) | 108-109 | -- |
| Share of recipients of state transfer payments | Recipients of state transfer benefits (social minimum income benefits) as a percentage of the main resident population in December 2018 | 118-119 | Includes basic cover for jobseekers (code of social law (SGB II)), basic cover in old age or in the event of reduced earning capacity (code of social law (SGB XII)), assistance for living expenses, and standard benefits under the Asylum Seekers Benefits Act. |

**Reference**

City Statistics. (2019). *Statistikatlas. Dortmunder Stadtteile* (Issue 215). <https://www.dortmund.de/media/p/statistik/pdf_statistik/veroeffentlichungen/statistikatlas/215_-_Statistikatlas_-_2019.pdf>, last accessed 09.08.2021
