# Supplementary material for: Associations between socio-spatially different urban areas and knowledge, attitudes, practices and antibiotic use: A cross-sectional study in the Ruhr Metropolis, Germany
Source: PLoS One. 2022 Mar 10;17(3):e0265204. doi: 10.1371/journal.pone.0265204 (PMC8912211; doi:10.1371/journal.pone.0265204)
Supplement: S1 File — (DOCX) [file pone.0265204.s003.docx]

**S1 File**

**Part A. All statements, questions and corresponding reply options
(in German and English language)**

**Knowledge statements**

Study participants were asked to indicate whether the following statements are (i) correct, (ii) wrong, (iii) “Don’t know” or (iv) could refuse to reply.

***Antibiotics***

| **English** | ***German*** |
| --- | --- |
| Antibiotics are effective against infections caused by bacteria. | *Antibiotika sind effektiv gegen Erkrankungen verursacht durch Bakterien* |
| Antibiotics are effective against infections caused by viruses. | *Antibiotika sind effektiv gegen Erkrankungen verursacht durch Viren.* |
| The flu and other common colds should be treated with an antibiotic. | *Die Grippe und andere Erkältungskrankheiten sollten mit einem Antibiotikum behandelt werden.* |
| Urinary tract infections should be treated with an antibiotic. | *Blasenentzündungen (Harnwegsinfekte) sollten mit einem Antibiotikum behandelt werden.* |
| Antibiotics kill naturally occurring bacteria on or in the body. | *Antibiotika töten auch natürlich vorkommende Bakterien auf oder in dem Körper ab.* |

***Antibiotic resistance***

| **English** | ***German*** |
| --- | --- |
| If an antibiotic is used too often or incorrectly, it can lose its effectiveness in the future. | *Wenn ein Antibiotikum zu oft oder falsch eingesetzt wird, kann es in Zukunft seine Wirksamkeit verlieren.* |
| The person that takes antibiotics will become resistant against antibiotics. | *Der Mensch, der Antibiotika einnimmt, wird resistent gegen Antibiotika.* |
| The use of antibiotics in agriculture can lead to lower effectiveness of antibiotics in humans. | *Der Einsatz von Antibiotika in der Landwirtschaft kann zu einer verminderten Wirksamkeit von Antibiotika bei Menschen führen.* |
| Antibiotic resistance threatens medical routine operations. | *Antibiotikaresistenzen gefährden medizinische Routine-Operationen.* |

**Attitude statements**

Study participants were asked to agree or disagree with the following statements on a five-point Likert scale: (i) strongly disagree, (ii) rather disagree, (iii) neutral, (iv) rather agree, (v) strongly agree or (vi) could refuse to reply.

| **English** | ***German*** |
| --- | --- |
| When I go to the doctor with a cold or flu, I expect an antibiotic so that I can get better quickly. | *Wenn ich mit einer Erkältungskrankheit oder Grippe zum Arzt gehe, erwarte ich ein Antibiotikum, damit es mir schnell besser geht.* |
| I request further information from my doctor, when s/he does not prescribe me an antibiotic. | *Ich frage nach, wenn mein Arzt mir kein Antibiotikum verordnet.* |
| When the symptoms subside after taking the antibiotic and I feel better, I can stop taking the antibiotic. | *Wenn die Beschwerden nach der Einnahme des Antibiotikums abklingen und ich mich besser fühle, kann ich das Antibiotikum absetzen.* |
| I prefer to have antibiotics in my cupboard at home for an emergency. | *Ich bevorzuge es, Antibiotika für einen Notfall zuhause im Schrank zu haben.* |
| It if fine to pass on stored antibiotics to relatives or friends, or to take them again myself, if they are similar symptoms. | *Es ist in Ordnung, wenn ich aufbewahrte Antibiotika an Verwandte oder Freunde weitergebe oder selber nochmal einnehme, sofern es ähnliche Symptome sind.* |

**Risk awareness statements**

Study participants were asked to agree or disagree with the following statements on a five-point Likert scale: (i) strongly disagree, (ii) rather disagree, (iii) neutral, (iv) rather agree, (v) strongly agree or (vi) could refuse to reply.

| **English** | ***German*** |
| --- | --- |
| Antibiotic resistance is already a global issue today. | *Antibiotikaresistenzen sind schon heute ein Problem auf der Welt.* |
| Antibiotic resistance is already an issue in Germany today. | *Antibiotikaresistenzen sind schon heute ein Problem in Deutschland.* |
| Antibiotic resistance can affect my families’ and my own health. | *Antibiotikaresistenzen können die Gesundheit von mir und meiner Familie beeinträchtigen.* |
| Antibiotic resistance is only an issue for people who take antibiotics regularly. | *Antibiotikaresistenz ist nur ein Problem für Menschen, die regelmäßig Antibiotika einnehmen.* |
| An antibiotic will remain effective against the same disease in the future. | *Ein Antibiotikum wird auch gegen die gleiche Krankheit in Zukunft noch effektiv sein.* |

**Handling practice questions**

Study participants could choose multiple times from the pre-determined reply options.

| **English** | ***German*** | **Reply options** |
| --- | --- | --- |
| Has any antibiotic ever been used in your household? | *Wurde in Ihrem Haushalt jemals ein Antibiotikum genutzt?* | - Yes - No - Don’t know - Not specified |
| From where do you get antibiotics in your household? | *Woher beziehen Sie Antibiotika in Ihrem Haushalt?* | - I used the leftovers from an old package - A relative/acquaintance gave it to me - From a doctor in the hospital - From a doctor in private practice - Don’t know - Not specified |
| How long are antibiotics used in your household? | *Wie lange wird ein Antibiotikum in Ihrem Haushalt angewandt?* | - Until the package is completely used - Until I feel better - According to the package insert - As recommended by the pharmacist - According to the doctor's instructions - Not specified |
| What happens to the leftover antibiotics in your household? | *Was passiert mit den Antibiotikaresten in Ihrem Haushalt?* | - Disposal at the pharmacy - Delivery to hazardous waste or mobile waste/hazardous material - Storage - Disposal via household waste - Disposal via the toilet - Everything has been used - Not specified |

**Antibiotic use questions**

| **English** | ***German*** | **Reply options** |
| --- | --- | --- |
| Have you taken an antibiotic in the past 12 months? | *Haben Sie in den vergangenen 12 Monaten ein Antibiotikum eingenommen?* | - Yes - No - Don’t know - Not specified |
| How often have you taken an antibiotic in the past 12 months? | *Wie oft haben Sie in den vergangenen 12 Monaten ein Antibiotikum eingenommen?* | Number of treatments (integer) |
| In what month(s) did you take an antibiotic? | *In welchem Monat/in welchen Monaten haben Sie ein Antibiotikum eingenommen?* | - January 2019 - February 2019 - March 2019 - April 2019 - May 2019 - June 2019 - July 2019 - August 2019 - September 2019 - November 2019 - December 2019 - January 2020 - February 2020 - March 2020 - Don’t know - Not specified |
| *For “Don’t know” only:* Can you narrow down the time period? | *Können Sie den Zeitraum eingrenzen?* | - Spring - Summer - Autumn - Winter |
| What did you take the antibiotic for? | *Wogegen haben Sie das Antibiotikum eingenommen?* | - Angina - Bronchitis - Diarrhoea - Cold - Fever - Joint/ tendon/ muscle inflammation - Flu - Sore throat - Urinary tract infection (cystitis) - Skin or wound infection - Headache - Lung infection - Ear infection - Prophylactic against secondary infections - Pharyngitis - Scarlet fever - Sniff - Tooth infection - Other complaints - Don’t know - Not specified |
| *For other complaints only:* What other health problems were they? | *Was für andere Beschwerden waren das?* | Write specific health issue (text) |

**Part B. All statements, questions and corresponding reply options
(in German and English language)**

**Table B.** Categorized coding of the outcome variables and covariates

| **Outcome variable** | **Grouping** |  | **Remarks** |
| --- | --- | --- | --- |
|  | **0** | **1** |  |
| Low knowledge | Correct | False or “Don’t know” |  |
| Attitudes contrary to common recommendations | Rather or strongly disagree | Neutral, rather or strongly agree |  |
| Low risk awareness^a^ | Rather or strongly agree | Neutral, rather or strongly disagree |  |
| Potential mishandling (index)^b^ | No mishandling practice reported | Any mishandling practice reported | Using an old package, stopping treatment when feeling better and storage of antibiotics at home were included |
| Self-reported antibiotic use | No antibiotic use reported | Antibiotic use reported |  |
| **Covariates** | **Reference** |  |  |
| Area | Area C | Area A, Area B |  |
| Age | NA |  | Continuous variable |
| Gender | Female | Male | One diverse person was removed |
| Immigration background | No | Yes | Defined as being an immigrant or descendant of immigrants |
| Family status | No partnership | In a partnership | Partner living in the same household |
| Education^c^ | Secondary (2) or post-secondary non-tertiary (3,4) | Tertiary (6,7,8) |  |
| Income | Below the national average | Equal to or above the national average | Average net income: 2,084 € per month in 2020 (statista, 2021) |
| Household income | Below the national average | Equal to or above the national average | Average net household income: 3,661 € per month in 2018 (Federal Statistical Office, 2020) |
| Occupational sector | Other | Health and social |  |
| Previous antibiotic use | No | Yes |  |

*^a^ This grouping was reversed for the statements on antibiotic resistance as an individual problem and future effectiveness; ^b^ Mishandling practices were too rare to examine individually, therefore all mishandling practices were summarized into a single index for each participant; ^c^ The International Standard Classification of Education (ISCED) was used. Corresponding codes provided behind the level of education in parentheses.*
